# Demonstration of a Low-Cost Multi-Pollutant Network to Quantify Intra-Urban Spatial Variations in Air Pollutant Source Impacts and to Evaluate Environmental Justice

**DOI:** 10.3390/ijerph16142523

**Published:** 2019-07-15

**Authors:** Rebecca Tanzer, Carl Malings, Aliaksei Hauryliuk, R. Subramanian, Albert A. Presto

**Affiliations:** 1 Department of Mechanical Engineering, Carnegie Mellon University, Pittsburgh, PA 15213, USA; 2 Center for Atmospheric and Particle Studies, Carnegie Mellon University, Pittsburgh, PA 15213, USA; 3 OSU-EFLUVE, CNRS, Université Paris-Est Créteil, 61 Avenue du Général de Gaulle, 94000 Créteil, France

**Keywords:** lower-cost sensor network, PM_2.5_, near-source

## Abstract

Air quality monitoring has traditionally been conducted using sparsely distributed, expensive reference monitors. To understand variations in PM_2.5_ on a finely resolved spatiotemporal scale a dense network of over 40 low-cost monitors was deployed throughout and around Pittsburgh, Pennsylvania, USA. Monitor locations covered a wide range of site types with varying traffic and restaurant density, varying influences from local sources, and varying socioeconomic (environmental justice, EJ) characteristics. Variability between and within site groupings was observed. Concentrations were higher near the source-influenced sites than the Urban or Suburban Residential sites. Gaseous pollutants (NO_2_ and SO_2_) were used to differentiate between traffic (higher NO_2_ concentrations) and industrial (higher SO_2_ concentrations) sources of PM_2.5_. Statistical analysis proved these differences to be significant (coefficient of divergence > 0.2). The highest mean PM_2.5_ concentrations were measured downwind (east) of the two industrial facilities while background level PM_2.5_ concentrations were measured at similar distances upwind (west) of the point sources. Socioeconomic factors, including the fraction of non-white population and fraction of population living under the poverty line, were not correlated with increases in PM_2.5_ or NO_2_ concentration. The analysis conducted here highlights differences in PM_2.5_ concentration within site groupings that have similar land use thus demonstrating the utility of a dense sensor network. Our network captures temporospatial pollutant patterns that sparse regulatory networks cannot.

## 1. Introduction

Poor air quality has deleterious health effects. Particulate matter with a diameter of less than 2.5 µm (PM_2.5_) dominates the human health burden from environmental exposures. PM_2.5_ is linked to cardiovascular disease and decreased life expectancy [1,2,3]. Other pollutants, including nitrogen dioxide (NO_2_), and sulfur dioxide (SO_2_) have health effects distinct from PM_2.5_. Exposure to NO_2_ and SO_2_ contributes to increases in cardiopulmonary mortality, cardiovascular disease, and respiratory disease [4]. SO_2_ and NO_2_ can be used to help attribute local enhancements in PM_2.5_ to emissions from coal-burning industries and traffic, respectively [5,6]. To quantify risks associated with exposure to these pollutants, it is necessary to measure and monitor their concentrations in the ambient environment.

Air quality monitoring has traditionally been conducted using sparsely distributed, expensive reference monitors. Traditional networks are good for capturing long-term temporal trends and inter-city differences [7], but they are generally too sparse to capture fine-scale within-city spatial variations [8]. Though there can be pollutant spatial variations at the sub-km scale [9], within this study, we define “fine-scale” as variations between different neighborhoods (~1 km^2^) throughout a large urban area. One way to improve spatial coverage of air pollutant monitoring is to deploy large networks of lower-cost sensors [10].

High spatial density networks of lower-cost monitors can be used to inform small-scale spatial variations in air pollution by providing real-time, on-the-ground measurements of air pollutants. However, previous studies using lower-cost sensors have usually focused on calibration or on calibration plus the deployment of a few nodes [11,12,13,14]. Many fewer papers demonstrate results from a large network of low-cost sensors [15,16]. In this study, we present results from a one-year deployment of a network of lower-cost monitors in Pittsburgh, PA, USA, focusing on 42 sensors in the network.

Widespread deployment of low-cost sensor networks also enables the investigation of environmental justice within a city. Environmental justice (EJ) is the fair treatment and meaningful involvement of all people regardless of race, color, national origin, or income with respect to the development, implementation and enforcement of environmental laws, regulations and policies [17]. The state of Pennsylvania defines a census tract with greater than or equal to 20% of the population living below the poverty line and/or greater than or equal to 30% of the population belonging to a minority group as an “EJ area” [18]. According to this definition, EJ areas are not necessarily areas that are currently experiencing environmental injustice. Rather, they are areas that have a high risk of experiencing environmental injustice as indicated by their socio-economic status. 

In this study, we utilize our dense network of air quality monitors to investigate whether the EJ areas in Pittsburgh do in fact have lower air quality in comparison to non-EJ areas. This definition of environmental injustice fits most closely with disparate exposure inequality. Disparate exposure inequality occurs when people belonging to a specific social group are more exposed to one or more environmental pollutants than they would be if the group was randomly distributed among the rest of the population [19]. 

Clark et al. used land-use regression (LUR) models to show that in the U.S. non-white (minority) populations often live in areas with higher air pollution [20,21]. They used a national LUR for NO_2_ to show that non-white populations are exposed to about 31% (3 ppb) higher mean concentrations of NO_2_, than white populations, primarily due to traffic emissions [21]. However, the exposure inequality trends identified by Clark et al. may not be identical in every city, as emission sources, land use, and population distributions might be idiosyncratic.

In this study, we use the RAMP (Real-time Affordable Multi-Pollutant sensor package) [22,23,24], a lower-cost monitor consisting of electrochemical gas sensors and PM_2.5_ nephelometers, to investigate spatial patterns in air pollution and exposure inequality in Pittsburgh. Sensor sites were distributed in such a way as to assess the variability in pollutant concentrations near known point sources and across urban and suburban/background locations. Using the low-cost sensors, we show that it is possible to detect enhancements of criteria pollutants that can be attributed to local sources like industry and traffic.

We also use the RAMP data to investigate exposure inequality in EJ and non-EJ areas as defined by the state of Pennsylvania. Previous work assessing air quality in EJ areas has typically used either national models that may not account for specific intra-urban pollutant variations [20,21] or used short-term (e.g., 1–3 week) intra-city measurements [25]. The expansiveness of our dense, low-cost sensor network, which was deployed for over a calendar year, captures pollutant measurements over various socio-economic areas within a city, allowing us to compare measurements taken in different EJ and non-EJ communities over a significant amount of time. The measurements lead us to conclude that socio-economic (EJ) factors do not necessarily determine PM_2.5_ exposures in different parts of Pittsburgh.

## 2. Materials and Methods 

### 2.1. Measurement Locations

**Figure 1:**
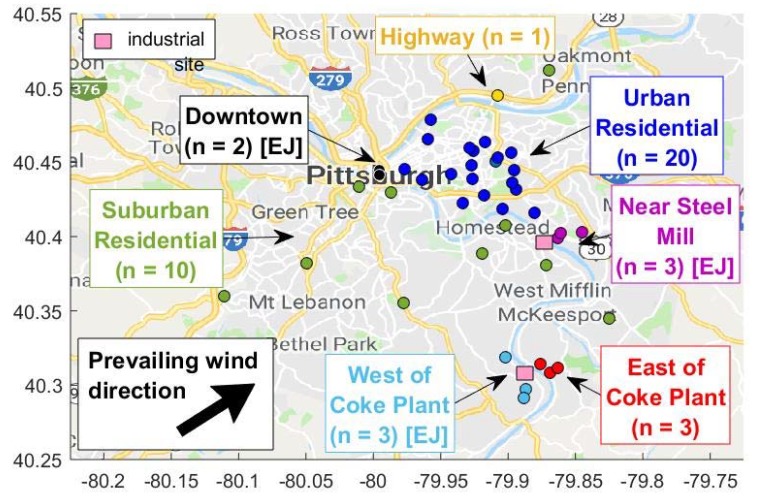
Map of the sampling domain. Dots indicate locations of 42 Real-time Affordable Multi-Pollutant sensor package (RAMP) monitors that were deployed throughout Pittsburgh. The sites were categorized into 2 Downtown sites, 20 Urban Residential sites, 10 Suburban Residential sites, 1 Near Highway site, 3 Near Steel Mill sites, 3 sites West of a Coke Plant, and 3 sites East of a Coke Plant based on traffic density, restaurant density, and proximity to industrial point sources (shown as pink squares). The prevailing wind direction is also shown; a wind rose is displayed in the Supplementary Information.

This paper focuses on data from forty-two RAMPs that were deployed throughout the greater Pittsburgh area in western Pennsylvania over April 2017–May 2018. Figure 1 shows the locations of RAMP sites throughout Pittsburgh and surrounding Allegheny County. RAMP sites cover a range of areas with varying land use and proximity to nearby emissions sources such as traffic, food cooking, and industry. The RAMP sites range from suburban residential sites with low traffic and low restaurant density, to downtown sites with high traffic and high restaurant density, to industrially influenced sites. RAMP sites also encompassed both EJ and non-EJ communities. While all of the RAMPs were nominally deployed for a year, the sites experienced various amounts of downtime due to sensor failures, power loses, and occasional returning of RAMPs to the Carnegie Mellon University campus for calibration [22]. Figure S1 in the Supplementary Information shows data coverage by season for each site.

The 42 sites were classified into seven categories: Downtown (*N* = 2 sites), Urban Residential (*N* = 20), Suburban Residential (*N* = 10), Highway (*N* = 1), Near Steel Mill (*N* = 3), West of Coke Plant (*N* = 3), and East of Coke Plant (*N* = 3). In Figure 1, similar RAMP locations, representative of particular micro-environments, are indicated with different colors. Sites were classified based on known land use. For Downtown, Urban and Suburban Residential, and Highway sites, the vehicle density within a 100-m radius and restaurant density within a 500-m radius of the site were used for classification. Values of vehicle and restaurant density were normalized by dividing the densities at each site by the maximum value across the entire sampling network for each variable. Downtown sites are located in the central business district and were in the top 30% of vehicle and restaurant densities. Urban Residential sites were located within the city limits and had moderate traffic density (below the 60th percentile) along with low restaurant density (within the first quartile). Suburban residential sites were those sites that were located outside of the city limits and experienced low vehicle and restaurant densities (within the first quartile). As the names suggest, the Urban and Suburban Residential sites were located in residential and mixed-use neighborhoods, typically at private residences or public schools. The Highway site was located 10 m from the edge of a limited-access highway.

Sites classified as Near Steel Mill, West of Coke Plant, and East of Coke Plant were defined by proximity to industrial point sources. The Near Steel Mill and East of Coke Plant sites were all within 1500 m of a steel mill and metallurgical Coke plant, respectively. These sites were east, and therefore generally downwind of, the respective point sources. West of Coke Plant sites were within 2000 m of the Coke plant in the generally upwind direction of the Coke plant.

The sites are listed in Table S1 in the Supplementary Information. Each site is assigned a numerical identifier that is used in subsequent figures. The site groupings are as follows: Downtown (Site 1–2), Urban Residential (3–22), Highway (23), Suburban Residential (24–33), Near Steel Mill (34–36), West of Coke Plant (37–39), and East of Coke Plant (40–42). The three sites Near the Steel Mill, three West of the Coke Plant, and the two Downtown sites are all classified as EJ communities by the state of Pennsylvania. To identify locations as EJ or not, census data was obtained. The latitude and longitude for each RAMP location were extracted and input into the EPA environmental justice screening tool, EJSCREEN. EJSCREEN was created by the U.S. EPA as a preliminary step in evaluating environmental justice issues [26]. The tool works in such a way that given a latitude and longitude it can output different socio-economic factors for the census block group in question. The census block group where each RAMP was located was identified and the percent of the population living below the poverty line and the percent of the population belonging to a minority group was extracted for each identified census block group.

### 2.2. Measurement Devices and Calibration

The Real-time Affordable Multi-Pollutant (RAMP) sensor package was used for this study. RAMPs were developed in a partnership between Carnegie Mellon University and SenSevere Limited Liability Company. Details about the RAMP monitoring package, including communication and data storage, are provided in Zimmerman et al. [24]. The RAMP data are recorded at a resolution of one data point approximately every 15 s, but for this study the data have been down-averaged to hourly mean concentrations. The RAMPs can measure up to four gaseous pollutants using electrochemical sensors from AlphaSense Ltd. The gaseous pollutants considered in this study are nitrogen dioxide (NO_2_, NO_2_-B43F) and sulfur dioxide (SO_2_, SO_2_-B4). NO_2_ and SO_2_ measurements were used as tracers for different PM_2.5_ sources (traffic and industrial point sources respectively). The RAMPs also included electrochemical sensors for measuring total oxidants (Ox, Ox-B431) and carbon monoxide (CO, CO-B41), as well as a nondispersive infrared (NDIR) CO_2_ sensor (SST CO_2_S-A) which also provided temperature and relative humidity data. Measurements from these additional three gaseous pollutant sensors are not used directly in this study.

Electrochemical gas sensors are commonly used in low-cost monitors because of their low cost to manufacture, selectivity, and simplicity [27,28]. These sensors consist of four electrodes. A redox reaction occurs between the working and counter electrodes when the sensor is exposed to the target pollutant. The reaction generates a potential difference which then can be correlated with concentrations of the pollutant. An auxiliary electrode in this four-electrode unit accounts for temperature and relative humidity effects. However, numerous studies have shown that assuming a linear relationship between sensor signal and concentration is insufficient to account for impacts of temperature, humidity, and sensitivity to species other than the target pollutant [10,11,13,22,24,29].

In this work, we follow the calibration method of Zimmerman et al. for NO_2_ [24]. This method uses (1) ambient collocation of RAMPs with EPA-grade reference monitors and (2) supervised machine learning algorithms to convert electrochemical sensor response to pollutant concentrations. Zimmerman et al. showed that a random forest machine learning algorithm provided the best performance for determining NO_2_ concentrations from RAMPs. The random forest calibrations yield precision and bias of ~25% for NO_2_. It has recently been shown that similar performance can be achieved using generalized calibration models rather than developing a unique calibration model for each RAMP [22]. Therefore, general calibration models were used for all of the RAMP NO_2_ data in this study. 

To calibrate the SO_2_ sensors, we collocated sixteen RAMPs with a reference grade SO_2_ monitor (Model 100A, Teledyne-API, San Diego, CA, USA) for three months at site 41. This site is <1 km east of the Coke plant and is often impacted by SO_2_ emissions from the plant. From the collocation a multi-linear regression calibration model was developed and applied to the SO_2_ sensors [23]. Details of the SO_2_ sensor collocation and calibration can be found in the Supplementary Information.

Each RAMP includes an optical PM_2.5_ monitor. Thirty-nine of the RAMPs used a Met-One Neighborhood PM Monitor (NPM) (Met One Instruments, Grants Pass, OR, USA) and the remaining 3 RAMPs used PurpleAir PA-II monitors. Table S1 in the Supplementary Information lists which sensor was placed at each site. These low-cost particulate matter sensors employ light scattering optical techniques instead of the traditional EPA regulatory PM monitoring techniques which include tapered element oscillating microbalances (TEOMs) and beta attenuation monitors (BAMs). Light scattering (also called nephelometry) is used in lower-cost sensors because they are cheap to manufacture, have low power requirements to operate, and have fast response times [30,31,32]. Light scattering devices typically are made up of an infrared emitting diode (IRED), a phototransistor, and focusing lens. When the particles pass through the sensor, they scatter light. The intensity of the scattered light is measured by a phototransistor and correlated with PM mass. Drawbacks to the light scattering technique include sensitivity to changes in temperature, relative humidity, particle composition, and size distribution [33,34,35].

To account for these effects, primarily the humidity artifact, we correct the as-reported PM_2.5_ mass concentrations to “BAM-equivalent” PM_2.5_ mass concentration. A detailed explanation of the correction method used here can be found in Malings et al. [36]. Briefly, we first correct for aerosol hygroscopic growth using temperature and relative humidity measured by each RAMP and the average particle composition measured in Pittsburgh. We then adjust the hygroscopic growth-corrected concentration to “BAM-equivalent” (values that can be directly compared to U.S. EPA standards) to account for aerosol size distribution effects by using a linear regression obtained by collocating the RAMPs with regulatory BAM monitors at sites 5 and 41.

This study considers data collected over a period of one year at each sampling site. However, the same RAMP was not deployed at each site for the entire study period. RAMPs are routinely brought back to our central reference site at the Carnegie Mellon University campus either for maintenance or for periodic calibration checks. As noted by Malings et al., the calibrations for gases measured by the RAMPs are robust for approximately 6–12 months, so the data used here are within the bounds for normal operation of these low-cost sensors [22]. In a separate paper, Malings et al. demonstrated that the PM_2.5_ measurements are robust for yearlong deployments [36].

## 3. Results and Discussion 

### 3.1. Intraurban PM_2.5_ Variability and the Impact of Point Sources

**Figure 2:**
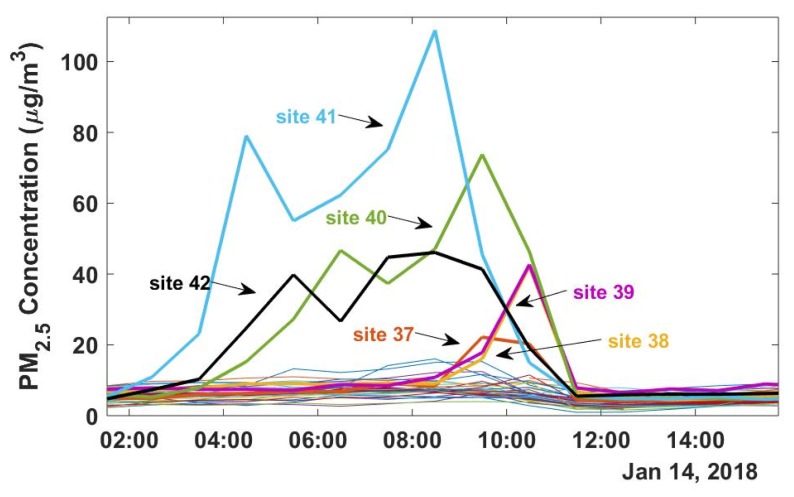
Example of a high PM_2.5_ event associated with local emissions. This figure is exemplary of periods of time throughout the study period when sites surrounding the Coke plant (indicated by thick lines) experienced elevated PM_2.5_ concentrations due to plant emissions while all other sites (thin lines) maintained background PM_2.5_ concentrations.

Although PM_2.5_ is largely regional [37], local point sources can be responsible for generating local spikes in PM_2.5_ mass. An example of pollution spikes due to local sources is shown in Figure 2, which compares 12 h of PM_2.5_ measurements at all 42 sites. PM_2.5_ concentrations are elevated relative to other sites at the three East of Coke Plant sites (40–42) starting at 2:00 a.m. on January 14th, 2018. PM_2.5_ concentrations at these sites increase from ~10 μg/m^3^ to as much as 100 μg/m^3^ over the course of several hours.

Prior to 8:00 a.m. the winds were blowing from the southwesterly direction; however, at 8:00 a.m. the winds shifted and began to blow from the northeasterly direction. This is accompanied by a drop in PM_2.5_ at sites 40–42 and a concurrent increase in PM_2.5_ at the West of Coke Plant sites (37–39), which were then downwind of the emissions source. The spikes measured at sites 37–42 were not observed at any other sites in the network, suggesting that this was a local enhancement due to emissions from the Coke plant.

**Figure 3:**
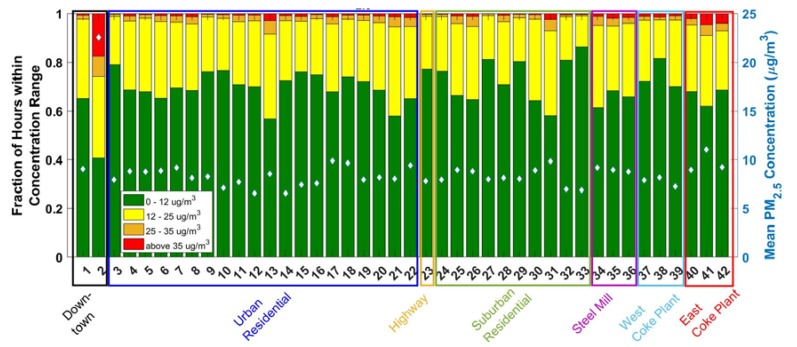
Average PM_2.5_ concentrations across the RAMP network. The bars show the fraction of hourly averaged PM_2.5_ measurements within each of four concentration ranges based on EPA and WHO regulatory cutoffs. Mean PM_2.5_ concentration is indicated within each bar as a white diamond.

Repeated instances of these types of spikes increase the long-term average concentrations at sites 40–42 which are predominately downwind of the Coke plant. Similarly, the Near Steel Mill sites, which are predominately downwind (east) of the steel mill (sites 34–36) also have higher long-term average PM_2.5_ concentrations than the sites that are upwind (west) of the steel mill in the suburban residential area (sites 27, 28, and 31). Figure 3 compares the PM_2.5_ measurements across the sampling network. The annual average concentration at each site ranges from 7.5 to 25.8 µg/m^3^, with the majority of sites having an average concentration less than the U.S. EPA annual standard of 12 μg/m^3^. 

The bar plot of each location in Figure 2 is subdivided into four categories: measurements where the hourly averaged PM_2.5_ concentration was (1) less than 12 µg/m^3^ (2) 12–25 µg/m^3^ (3) 25–35 µg/m^3^ and (4) greater than or equal to 35 µg/m^3^. These cutoffs were chosen based on EPA and World Health Organization (WHO) daily and annual average PM_2.5_ standards. The 25 µg/m^3^ level is the WHO 24-h exposure standard; if concentrations are above this threshold for more than 24 h, that would be hazardous according to the WHO. With the exception of site 2, which is described in more detail below, all sites had PM_2.5_ lower than 12 µg/m^3^ for over 57% of hours, and concentrations were less than 25 µg/m^3^ for over 91% of all hourly data.

Sites that had relatively higher percentages of hours with measured PM_2.5_ above 25 μg/m^3^ were further investigated. Sites with elevated annual average PM_2.5_ are generally impacted by local enhancements, and therefore experience concentrations exceeding 25 µg/m^3^ more often than sites that are far from either point sources or areas of high traffic density. Site 2 has the highest percent of hours above 25 µg/m^3^ (25.9%). This anomalously high occurrence of elevated PM_2.5_ concentrations can be attributed to the fact that site 2 is located downtown in a street canyon approximately ten meters away from the exhaust of a restaurant with a wood-fired pizza oven. These cooking emissions drive the elevated PM_2.5_ concentrations for site 2.

The three East of Coke Plant sites (40–42) also experienced elevated frequencies of hourly PM_2.5_ concentrations exceeding 25 µg/m^3^. They are located within 1500 m of the coke plant, in the predominantly downwind direction. These sites experienced PM_2.5_ concentrations over 25 µg/m^3^ for 4.8%, 8.2%, and 6.9% of the sampling period. In contrast, across all other sites (excluding site 2), concentrations above 25 µg/m^3^ occur only 3.2 ± 1.7% of the time. Sites 41 and 42 were more than two standard deviations higher than this average, while site 40 was on the upper end of that range. 

Two of the sites East of the Coke Plant (41 and 42) experience higher PM_2.5_ concentrations than the third site. The presence of these differences points to the utility of dense lower-cost networks of air quality monitors, as a single, expensive regulatory monitor would be incapable of capturing this level of fine-scale spatial variability. 

Large differences in PM_2.5_ concentration exist between different site groupings. The difference in PM_2.5_ concentrations upwind and downwind of the Coke plant illustrate sharp PM_2.5_ gradients that can result from industrial point sources. The three West of Coke Plant sites (37–39), which are similarly close (1–2 km) to the Coke plant as the East of Coke Plant sites, have PM_2.5_ greater than 25 µg/m^3^ only 2.7%, 2.6%, and 2.7% of the time, respectively. This is similar to the Urban and Suburban Residential sites. 

We can also use Figure 3 to assess how the frequency of elevated PM_2.5_ concentration varies in EJ versus non-EJ communities. Sites 37–39 are classified as EJ communities, whereas sites 40–42 are non-EJ communities. However, sites 40–42 experience a higher frequency of hours with elevated PM_2.5_ concentrations than sites 37–39. The low-cost sensor network in this region is able to detect influences from point sources on a finely resolved spatial scale in a way that illuminates differences in EJ and non-EJ communities.

Figure 3 shows all of the data collected during our study period at each site. Additional plots separating the data by season are shown in Figure S2 in the Supplementary Information. PM_2.5_ concentrations vary seasonally, with lower concentrations in the spring than in the other three seasons; mean PM_2.5_ concentration in the fall, winter, spring and summer were 11.2, 10.5, 8.7, and 13.9 µg/m^3^ respectively. Across the network, PM_2.5_ concentrations exceeded 25 µg/m^3^ for only 1.5% of hours in the spring, versus 5.25%, 4.35%, and 5.00% in the summer, fall, and winter, respectively. The spatial pattern of high PM_2.5_ concentrations, however, remains consistent from season to season, largely driven by local emissions. The restaurant impacted site 2 always has the highest PM_2.5_ concentration, and sites 40–42 East of the Coke Plant have more frequent instances of PM_2.5_ greater than 25 µg/m^3^ than West of Coke Plant or Residential sites.

The coefficient of divergence (COD) is a metric that can be used to determine the significance of PM_2.5_ concentration differences between sites. The COD is computed using Equation (1) for each pair of sites.

(1) $$\mathrm{COD}=\sqrt{\frac{1}{N}\overset{N}{\underset{i=1}{\sum }}{\left(\frac{x_{iA}-x_{iB}}{x_{iA}+x_{iB}}\right)}^{2}}$$

*N* is the number of paired observations, *x_iA_* is the measurement at time period *i* for site A, and *x_iB_* is the measurement at the time period *i* for site B, where each time period *i* is one hourly averaged PM_2.5_ measurement. A threshold of 0.2 is typically used to identify pairs of sites that are significantly different (COD > 0.2) from sites that are similar (COD < 0.2) [25,38]. 

During our evaluation of these low-cost sensors by collocation with a reference monitor, the majority of the sensor pairs showed a COD below 0.2. Figure S3 in the Supplementary Information shows the results of analysis conducted on 48 RAMPs that were collocated at site 7. While 6 pairs of RAMPs at the collocation had CODs over 0.2, the remaining 1122 pairs of RAMPs showed a COD less than 0.2. Hence, we expect that when the sensors are deployed, CODs greater than 0.2 signify actual differences in PM_2.5_ concentration and are not due to sensor noise.

**Figure 4:**
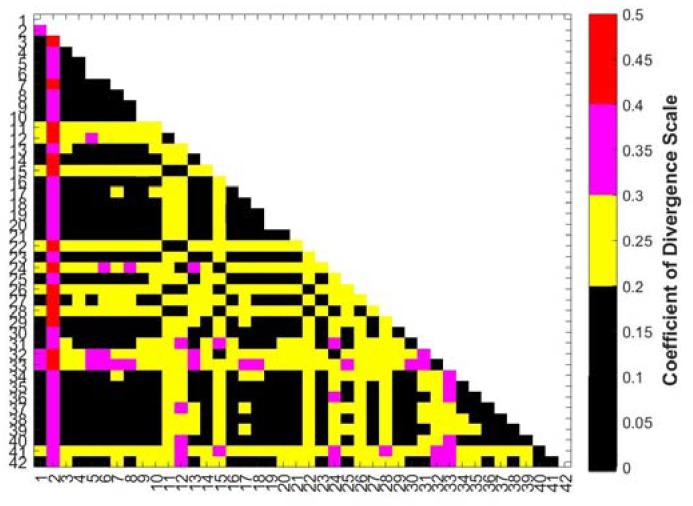
Pairwise hourly coefficients of divergence (COD) for PM_2.5_. As the COD for the majority of pairs is greater than 0.2 there exists heterogeneity on an hourly basis between the sites.

Figure 4 shows the COD for hourly averaged PM_2.5_ concentrations between each pair of sites. The COD suggests that there is significant spatial heterogeneity across the RAMP network on an hour-to-hour basis. More than half of the pairwise COD values are greater than 0.2. The analyses of Figure 2 and Figure 3 above focused on the most extreme differences (e.g., site 2 versus all other sites). However, the COD matrix in Figure 4 shows that there are also subtle, but meaningful, differences between many more sites, even those within a site class. 

Although we are not able to quantify all of the sources of variability that drive CODs to be greater than 0.2 between site pairs, one source of variability is emissions from local point sources. The CODs between the East of Coke Plant sites and sites not impacted by point sources are for the most part greater than 0.2. As shown in Figure 2, emissions plumes can impact different sets of sites at different times, depending on meteorological conditions. Plumes can also advect downwind, and there are examples in our dataset of plumes starting near the Coke plant that eventually impact some, but not all, of the Urban Residential sites. This time lag (in addition to dilution) while plumes travel from one area to another can cause differences in PM_2.5_ concentrations measured on an hourly basis between sites and thus lead to significant differences in hourly averaged PM_2.5_.

### 3.2. Multi-Pollutant Patterns

The gaseous pollutants measured by the RAMPs offer insight into the sources driving the inter-site differences in PM_2.5_ concentrations. In this section, we use NO_2_ as an indicator for traffic emissions and SO_2_ as a marker for industrial emissions to aid in describing the PM_2.5_ trends at various site types. 

**Figure 5:**
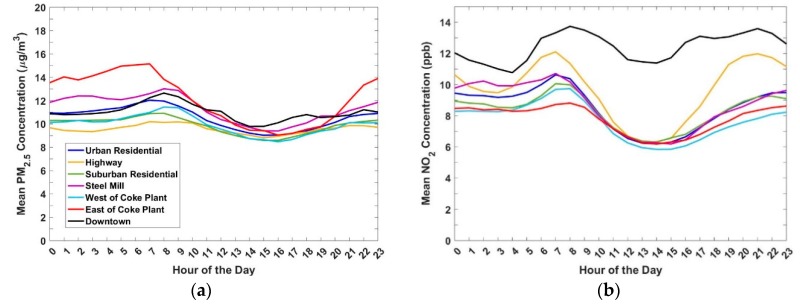
(**a**) Hourly averaged diurnal patterns of PM_2.5_ within each site group. Downtown site 2, which is impacted by emissions from a nearby restaurant, is not included. (**b**) Mean diurnal patterns of NO_2_ for each of the site groups. All sites were used because the restaurant near site 2 is not a major NO_2_ source.

Figure 5a shows the average diurnal pattern of PM_2.5_ for each site group. The diurnal patterns at each of the seven study areas (Downtown, Urban Residential, Highway, Near Steel Mill, Suburban Residential, West of Coke Plant, and East of Coke Plant) were determined by averaging the measurements taken at each respective hour of the day for all of the locations within each study area. For the Downtown PM_2.5_ diurnal we ignored site 2, and therefore only site 1 was used. As described above, site 2 is heavily impacted by emissions from a nearby restaurant and therefore may not be representative of the broader downtown area. For the rest of the site groups all sites within the group were included in calculating the diurnals. 

Some common trends are observed across the sampling domain. PM_2.5_ concentrations increase in the morning at most sites (~7–9 a.m.). This general trend is mirrored by NO_2_ (Figure 5b), which also exhibits a domain-wide increase during the morning rush hour. The concurrent morning peaks in PM_2.5_ and NO_2_ are indicative of rush hour traffic emissions, combined with low atmospheric mixing height. PM_2.5_ concentrations reach a minimum around 3–4 p.m. as the atmosphere becomes more well mixed. There is no early evening PM_2.5_ enhancement during the evening rush hour at most of the sites.

Figure 5 shows that multi-pollutant concentration patterns, and therefore exposure, change throughout the day. In the evening through early morning the East of Coke Plant and Near Steel Mill sites have the highest mean PM_2.5_ concentrations. People who live in these areas are presumably at home during these times, and therefore likely to be exposed to elevated PM_2.5_ relative to other areas in our study domain. However, during the day, Downtown has the highest PM_2.5_ concentrations. This means that someone who lives in one of the areas East of the Coke Plant or Near the Steel Mill but works Downtown could have higher exposures than someone who both lives and works in one of the industrially influenced areas. This has important implications for public health; it may not be enough to incorporate one’s residence in exposure assessment, since workday exposures in downtown or other commercial areas may be significantly different than in residential neighborhoods.

There are differences in the diurnal trends and in the absolute concentrations between site groups. For example, all of the sites except for Downtown exhibit a sharp drop in PM_2.5_ concentrations after the morning rush hour. This is driven by a decrease in the traffic source and an increase in atmospheric mixing height. In Downtown; however, PM_2.5_ concentrations decrease more gradually throughout the workday. This can be attributed to elevated traffic emissions throughout the day relative to other areas, along with contributions from street canyon effects and restaurant cooking [39]. The measured NO_2_ concentrations suggest that traffic is a driver for the excess PM_2.5_ in downtown. NO_2_ in Downtown remains high during the day compared to other site groups and is the only site group (with the exception of the Highway site) that shows an afternoon rush hour peak in NO_2_.

The East of Coke Plant sites and Near Steel Mill sites experience some of the highest PM_2.5_ concentrations at all times of the day. The enhancements in mean PM_2.5_ concentration at the East of Coke Plant sites and Near Steel Mill sites are larger in the late evening through early morning than the enhancements observed at any of the other sites. The individual contributions of micrometeorology and higher industrial emissions at night cannot be separated with this dataset and should be investigated in future work. In contrast to the elevated PM_2.5_, NO_2_ concentrations at these sites during the day are similar to the Urban and Suburban Residential sites; hence, unlike Downtown, traffic is likely not a significant contributor to the higher PM levels in the area. On the other hand, SO_2_ concentrations at the Near Steel Mill and East of Coke Plant sites are frequently elevated above background levels. This suggests that industrial emissions play an important role.

**Figure 6:**
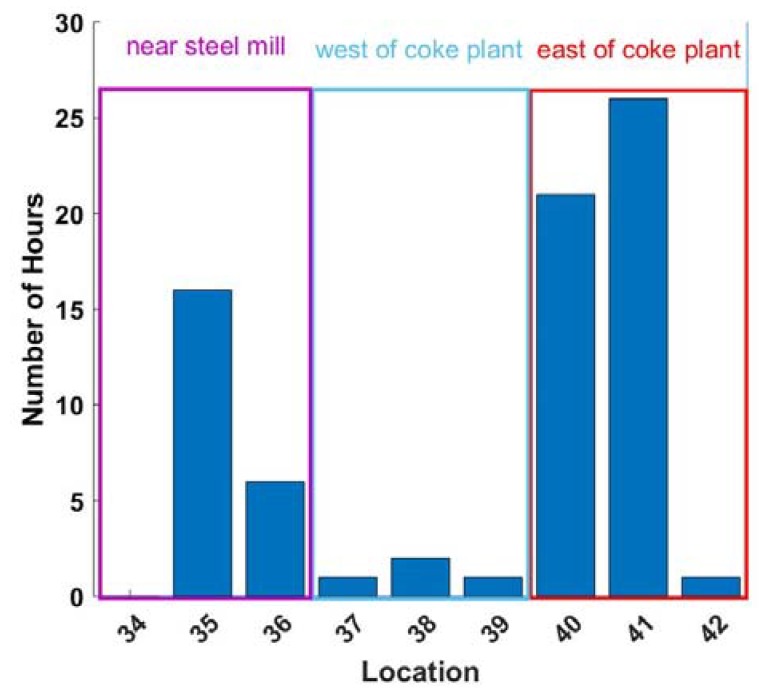
Frequency of high SO_2_ concentrations. Bars show the number of hours with SO_2_ greater than 50 ppb from November 2017 through May 2018 at the nine sites located near the coke plant and steel mill. Sites downwind of the coke plant have the most frequent occurrences of high SO_2_.

SO_2_ measurements were used as a tracer for industrial emissions. Figure 6 shows the number of hours for which SO_2_ concentrations exceeded 50 ppb (99.8th percentile of SO_2_ measurements) at the nine sites near the steel mill and Coke plant. Instances of high SO_2_ were most frequent at the East of Coke Plant and Near Steel Mill sites (which are usually downwind), suggesting that emissions from these sources contribute to the occasions of high PM_2.5_ shown in Figure 3. 

We investigated correlations between background-corrected PM_2.5_ concentration and SO_2_ concentration to test whether these pollutants have a common source. The background-corrected PM_2.5_ concentration was obtained by subtracting the PM_2.5_ measured at Urban Residential site 5 from the measured PM_2.5_ concentration at the source influenced sites. Background-corrected PM_2.5_ concentration and SO_2_ concentrations were normalized for each site and scatter plots for each site are shown in the Supplementary Information (Figure S4). The mean R^2^ value for correlation between PM_2.5_ and SO_2_ for the nine source impacted sites is 0.32 (ranging from 0.16–0.56), compared to near zero correlation at the background sites (R^2^ at site 5 = 0.03). In particular, variations in SO_2_ explain about 40% of the variation in PM_2.5_ at sites 41 and 42 (East of the Coke Plant sites), which is significantly higher than any of the other source impacted sites. As observed earlier, these two sites also saw significantly higher PM_2.5_ than the Urban and Suburban Residential sites. This suggests that the elevated PM_2.5_ concentrations at sites East of the Coke plant are more heavily influenced by emissions from the Coke plant when compared to the other source impacted sites in the area, and even among sites east (downwind) of the Coke plant, there can be differences that are revealed by a high-density sensor network. 

The West of Coke Plant sites have lower SO_2_ than the East of Coke Plant sites, echoing the results for PM_2.5_ because these sites are often upwind of the source. Furthermore, a regulatory SO_2_ reference monitor located at site 5 (Urban Residential) recorded zero hours of SO_2_ concentration above 50 ppb during the study period. The overall story is that the industrial emissions drive the elevated PM_2.5_ concentrations in the areas downwind of the Coke and steel plants, not traffic.

Figure 6 also shows that there is heterogeneity within the site classes. One of the Near Steel Mill sites (site 34) never experienced SO_2_ greater than 50 ppb during the study period. Likewise, site 42 had fewer instances of high SO_2_ than sites 40 and 41. Although there are broad similarities in sites with similar land use and nearby sources, there is variability even within site classes. The COD for SO_2_ for all site pairs between the nine sites near industrial facilities was greater than 0.2. A plot of the pairwise COD for SO_2_ at these nine sites is found in the SI, Figure S5. The heterogeneity between SO_2_ concentrations within site groupings further demonstrates the utility of a high-density multi-pollutant network.

### 3.3. Exposure Inequality and Environmental Justice

**Figure 7:**
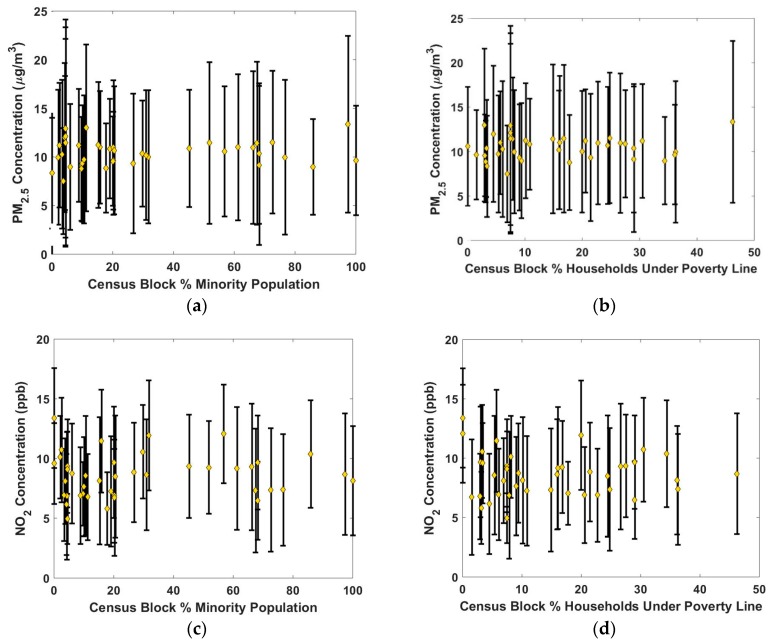
Environmental justice analysis showing mean (yellow diamond) and standard deviation (whiskers) at each site. Site 2 was not included in the PM_2.5_ analysis due to the impact of the local restaurant emissions at that site. (**a**,**b**) show the lack of correlation between PM_2.5_ concentrations and percent of the population who (**a**) belong to a minority group or (**b**) are living below the poverty line. (**c**,**d**) similarly show the lack of correlation between NO_2_ concentrations and the same two socio-economic variables.

Figure 7 examines exposure inequality and environmental justice of PM_2.5_ and NO_2_ as a function of two socio-economic variables: percent of the population living below the poverty line and percent of the population belonging to a minority group. Although there are numerous socio-economic factors available for assessing environmental justice, this study only analyzes these two factors as they are the indicators for environmental justice regions in the state of Pennsylvania. The mean PM_2.5_ concentration for all of the non-EJ sites is 10.3 µg/m^3^ (standard deviation = 1.5 µg/m^3^) and the mean PM_2.5_ concentration for all of the EJ sites is 10.6 µg/m^3^ (standard deviation = 1.0 µg/m^3^), which suggests no significant difference in PM_2.5_ concentrations based on EJ status of the census block group.

Spearman’s rho, a non-parametric measure of rank correlation, can be used to test the relationship between two variables. A Spearman’s rho with an absolute value of less than 0.20 is indicative of very weak correlation between the variables, only above 0.60 is the correlation considered strong. The Spearman’s rho between mean PM_2.5_ concentration at a site and percent of the population living below the poverty line in the census block group is 0.05. The Spearman correlation between mean PM_2.5_ and percent of the population belonging to a minority group is similarly low (0.01). This means that the relationship between mean PM_2.5_ concentration and socioeconomic (EJ) variables cannot be described by a monotonic function; PM_2.5_ concentration does not increase with increasing EJ indicators. 

The Spearman’s rho between the mean NO_2_ concentrations at the RAMP sites and the socio-economic variables is similarly low; 0.01 and 0.06 when comparing mean NO_2_ at a site to percent of the population living under the poverty line and percent minority group, respectively. The mean NO_2_ for EJ sites was 8.85 ppb (standard deviation = 1.58 ppb) while the mean NO_2_ concentration for non-EJ sites was 8.32 ppb (standard deviation = 2.00 ppb). In other words, NO_2_ concentrations are not systematically higher in EJ communities than non-EJ communities within our study domain.

In contrast to our findings, Clark et al. showed strong correlation between EJ communities and elevated NO_2_ concentrations and reported that on a national scale the population weighted mean NO_2_ concentrations for non-whites were 5 ppb higher than for whites in 2000 and 2.9 ppb higher in 2010 [21]. There are several possible explanations for the disagreement of our results with those of Clark et al. One potential explanation is methodological. Clark et al. used a national land use regression model estimate of NO_2_ whereas we use a dense network of sensors within the county. We have 42 monitors running in the relatively small study domain, while Clark et al. used a model that was trained on the national EPA monitoring system that only includes two monitors in our domain. Additionally, Clark et al. reported on average trends throughout the nation. There is no requirement that each individual city follow these trends; due to different socio-economic factors, Pittsburgh may not follow the national average trend. For example, several of the Urban Residential sites are located in neighborhoods that are a mix of middle to upper income families and college students. The student population increases the percent of non-white population while decreasing the average income of the areas. There may also be nuanced differences with the ways that minority populations were defined in each study that may have impacts on the results. For example, in our study we simply defined percent minority population as the non-white portion of the population. If we were to break the non-white portion of the population into different subgroups there may be different patterns that arise in our results. Furthermore, many of the EJ areas, as defined by race and income, are typically upwind of industrial facilities and thus less impacted by these emissions. 

## 4. Conclusions

A dense network of over 40 lower-cost monitors was deployed within the city of Pittsburgh and surrounding areas in Allegheny County. The dense sensor network was able to detect significant differences in PM_2.5_ concentration between groups of sites within the study domain, and also between sites within a site group with similar characteristics. NO_2_ was used as a tracer for traffic emissions and SO_2_ was used as a tracer for industrial emissions. Downtown and near Highway sites experienced elevated PM_2.5_ and NO_2_ concentrations that were dominated by traffic emissions. Sites downwind of industrial sources such as the Near Steel Mill sites and East of Coke Plant sites experienced elevated PM_2.5_ concentrations influenced by industrial point sources, indicated by higher SO_2_ levels. No relationship was found linking two socio-economic variables to elevated PM_2.5_ or NO_2_ concentrations within our sampling network. 

Our analysis demonstrates the value of a dense sensor network. Our network is able to capture temporospatial pollutant patterns that cannot be resolved by the sparse network of regulatory monitors. We grouped our sensors into seven categories and observed significant variations both within and between categories. Even if the regulatory monitoring network had one site in each of our seven land-use-based categories (and it does not), it would not be able to capture all of the spatial variations that we present here. Coupling measurements of PM_2.5_ and gases allows us to attribute the observed temporospatial pollutant patterns to specific source classes, which demonstrates the benefit of multipollutant sensor networks. 

The approach we use here could easily be replicated in other cities. While the mix of sources may be different—for example, the coke plant is somewhat unique to our sampling domain—networks of multi-pollutant sensors should be capable of capturing pollutant patterns and attributing them to traffic versus other sources.

## Supplementary Materials

The following are available online at https://www.mdpi.com/1660-4601/16/14/2523/s1, Table S1: RAMP Locations, Figure S1: Seasonal Data Coverage, Figure S2: Seasonal Concentrations of PM_2.5_, Figure S3: Variability Among Co-Located RAMPs, Figure S4: Relationships between Excess PM_2.5_ and SO_2_, Figure S5: Variability of SO_2_. Figure S6: Wind rose.
