# Supplementary material for: Demonstration of a Low-Cost Multi-Pollutant Network to Quantify Intra-Urban Spatial Variations in Air Pollutant Source Impacts and to Evaluate Environmental Justice
Source: Int J Environ Res Public Health. 2019 Jul 15;16(14):2523. doi: 10.3390/ijerph16142523 (PMC6678618; doi:10.3390/ijerph16142523)
Supplement: Supplementary file 1 [file ijerph-16-02523-s001.pdf]

Article

Demonstration of a Low-Cost Multi-Pollutant Network to Quantify Intra-Urban Spatial Variations in Air Pollutant Source Impacts and to Evaluate Environmental Justice

Rebecca Tanzer [1,2], Carl Malings [2,3], Aliaksei Hauryliuk [1,2], R. Subramanian [2,3] and Albert A. Presto [1,2,*]

Supplemental Information (SI)

SO_2 Calibration

In this study we collocated sixteen RAMPs with a reference grade SO_2 monitor (Teledyne T100A) for three months at site 41. This site is less than 1 km east of the coke plant. SO_2 is a known byproduct of coke production. Hourly averaged SO_2 concentrations ranged from ~0 to greater than 100 ppb during the collocation period at site 41, which provided sufficient dynamic range for training calibration models.

Following Zimmerman et al. and Malings et al., we developed both multi-linear regression (MLR) and machine learning based neural network (NN) calibrations for SO_2. The inputs for the MLR model are net SO_2 signal, temperature, and relative humidity measured by the RAMP. The inputs for the NN model were net signal for five gaseous pollutant sensors (SO_2, CO, NO_2, O_3, and CO_2), temperature, and relative humidity.

At the calibration site both the MLR and NN models performed well, with R^2 of 0.60 and 0.75, respectively, for calibration testing. However, when the models were applied to a RAMP at a second collocation site with a reference monitor (site 35) the performance of the NN model drastically dropped (R^2 = 0.11). The MLR model on the other hand maintained acceptable performance (R^2 = 0.54). This decrease in performance by the more complex NN calibration model may be attributed to an overtraining of the model on the source mixture at site 41. This in turn led to less transferability of the NN-based calibration. Therefore, the MLR calibration model was used here.

Figures

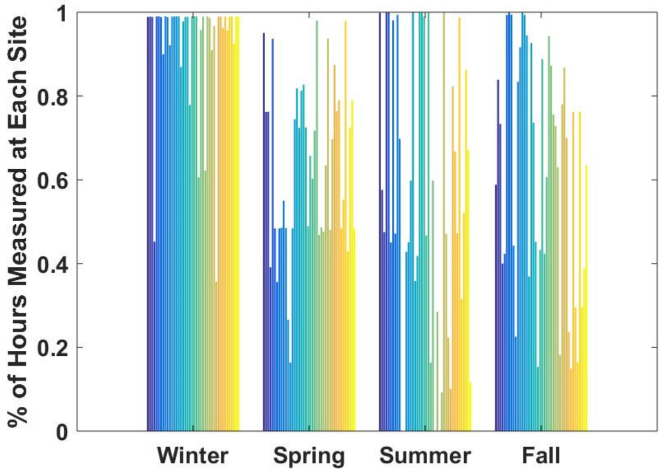

Figure S1. Seasonal Data Coverage. 42 vertical bars are shown for each RAMP in each season. The heights of the bars indicate the percent of hours measured in each season at each of the 42 RAMP sites. These 42 sites were sub selected down from an original list of 77 RAMPs to include only sites that were collecting data for at least half of a year (4380 h) during the study period. Good seasonal coverage was obtained using this dataset. The median coverage per season per RAMP was 69% of the season covered.

Table S1. RAMP Locations.

Identifier	Area	Type of PM Monitor
1	Downtown	MetOne
2	Downtown	MetOne
3	Urban Residential	MetOne
4	Urban Residential	MetOne
5	Urban Residential	MetOne
6	Urban Residential	MetOne
7	Urban Residential	MetOne
8	Urban Residential	MetOne
9	Urban Residential	MetOne
10	Urban Residential	MetOne
11	Urban Residential	MetOne
12	Urban Residential	MetOne
13	Urban Residential	MetOne
14	Urban Residential	MetOne
15	Urban Residential	MetOne
16	Urban Residential	MetOne
17	Urban Residential	MetOne
18	Urban Residential	MetOne
19	Urban Residential	MetOne
20	Urban Residential	MetOne
21	Urban Residential	MetOne
22	Urban Residential	MetOne
23	Highway	MetOne
24	Suburban Residential	MetOne
25	Suburban Residential	MetOne
26	Suburban Residential	Purple Air

27	Suburban Residential	MetOne
28	Suburban Residential	Purple Air
29	Suburban Residential	MetOne
30	Suburban Residential	MetOne
31	Suburban Residential	MetOne
32	Suburban Residential	MetOne
33	Suburban Residential	Purple Air
34	Steel Mill	MetOne
35	Steel Mill	MetOne
36	Steel Mill	MetOne
37	West of Coke Plant	MetOne
38	West of Coke Plant	MetOne
39	West of Coke Plant	MetOne
40	East of Coke Plant	MetOne
41	East of Coke Plant	MetOne
42	East of Coke Plant	MetOne

Each site was assigned to an area grouping and a number from 1 to 42. The last column indicates whether the RAMP's $PM_{2.5}$ monitoring device was a MetOne nephelometer or a PurpleAir laser sensor.

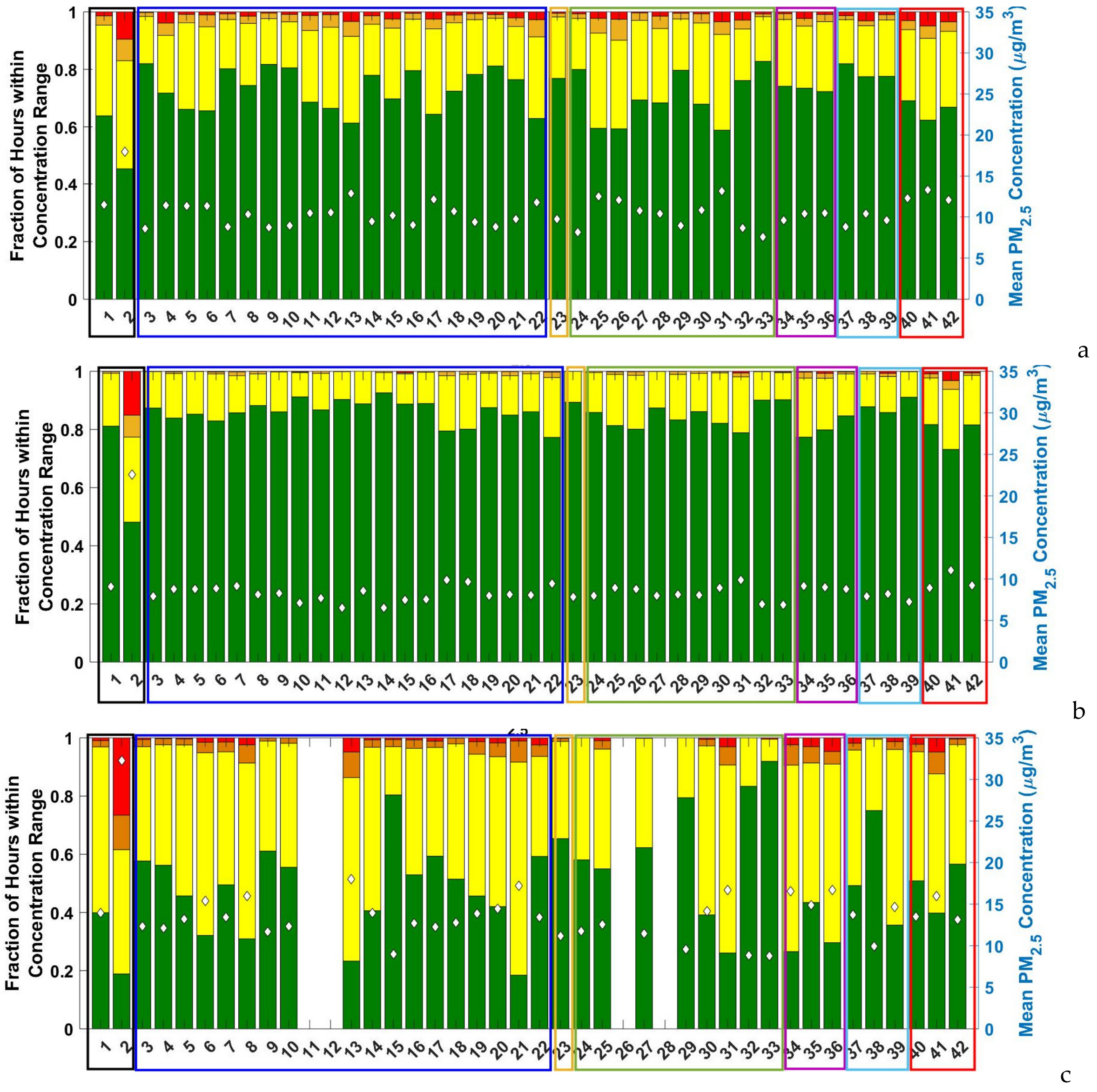

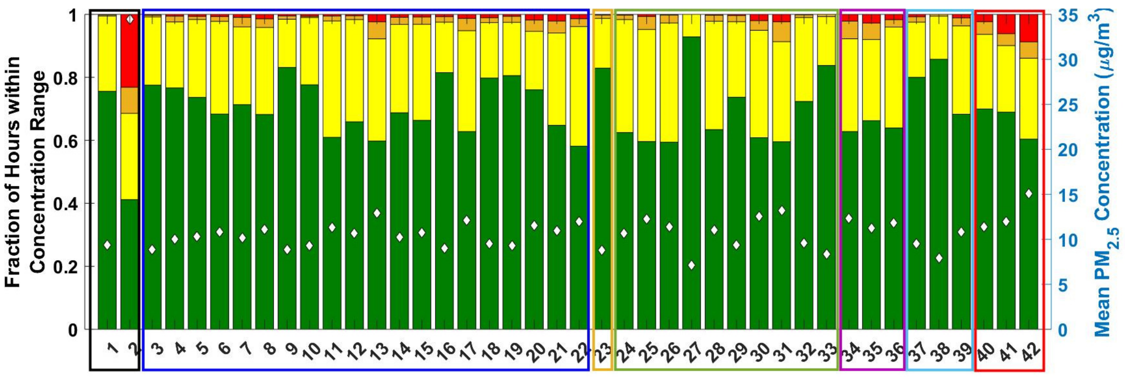

d

Figure S2. Seasonal Concentration of PM2.5. Green bars are fraction of hours with PM2.5 concentration less than 12 μg/m^3, yellow bars (12-25) μg/m^3, orange bars (25-35) μg/m^3, and red bars are fraction of hours greater than or equal to 35 μg/m^3. The data from Figure 3 in the text was subdivided by season to differentiate the seasonal differences in PM2.5 at each site for (**a**) winter, (**b**) spring, (**c**) summer, and (**d**) fall.

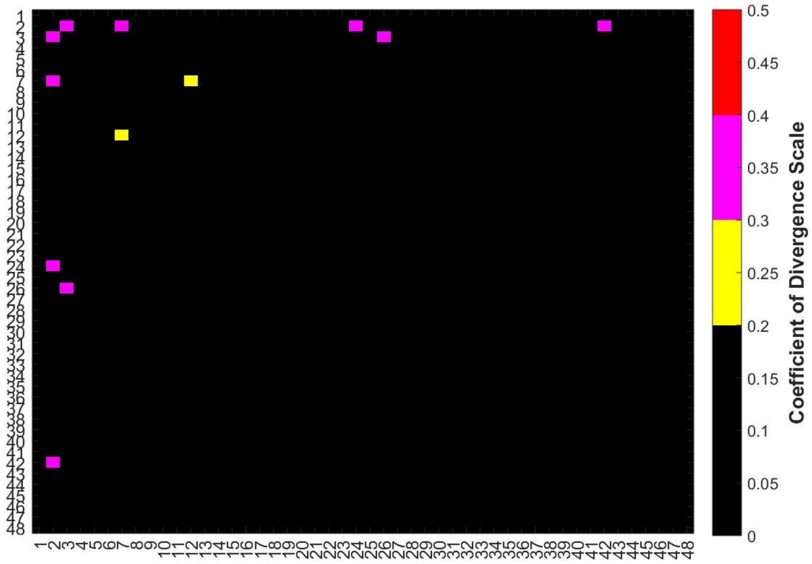

Figure S3. Variability among co-located RAMPs. 48 RAMPs were collocated at site 7. The hourly averaged COD for each pair of sensors at this collocation was calculated and all but 6 pairs had insignificant differences in measurement from the other sensors. From this we conclude that the differences in COD that are shown in Figure 4 can be attributed to actual differences in measured PM2.5 and not sensor noise.

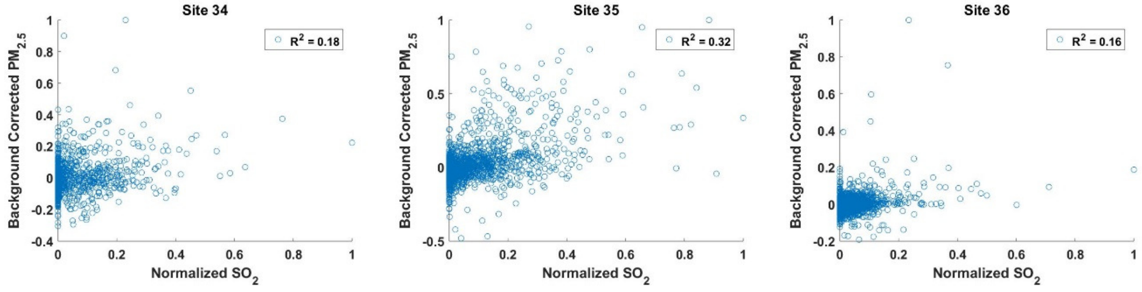

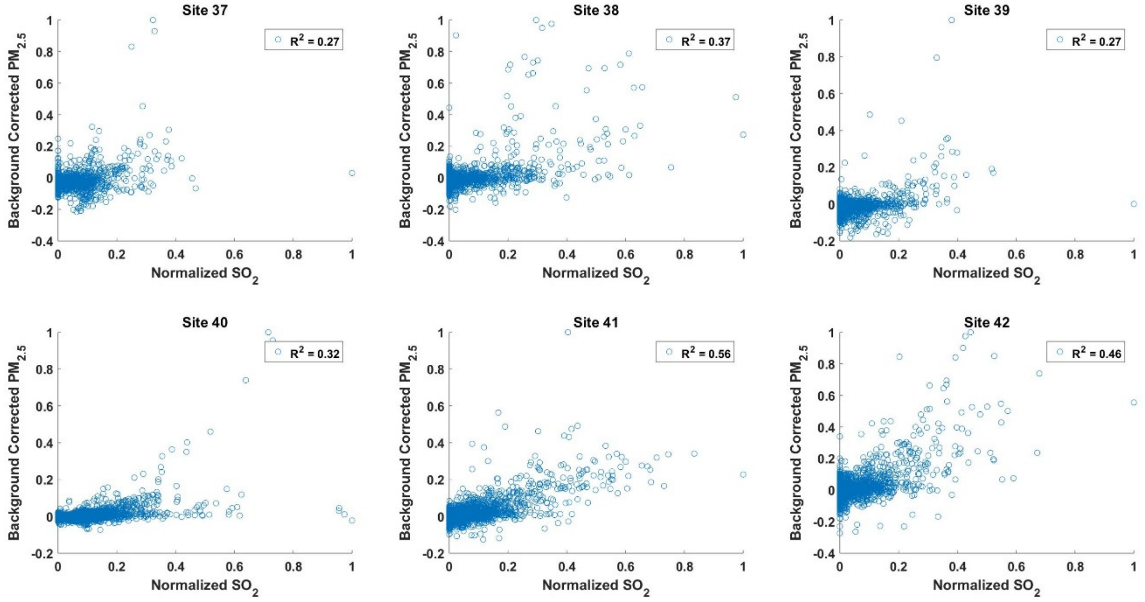

Figure S4. Relationships between excess $PM_{2.5}$ and SO_2. The background corrected $PM_{2.5}$ concentration was calculated for each site by subtracting the $PM_{2.5}$ concentration measured at an Urban Residential site (site 5) from the source influenced sites. The hourly averaged background corrected $PM_{2.5}$ concentrations were then normalized and correlated to the normalized SO_2 measurements at each of the sites near industrial facilities. Two sites downwind of the coke plant (sites 41 and 42) show the strongest correlation between $PM_{2.5}$ and SO_2 indicating that the elevated $PM_{2.5}$ concentrations at those locations are heavily influenced by SO_2 carrying industrial emissions.

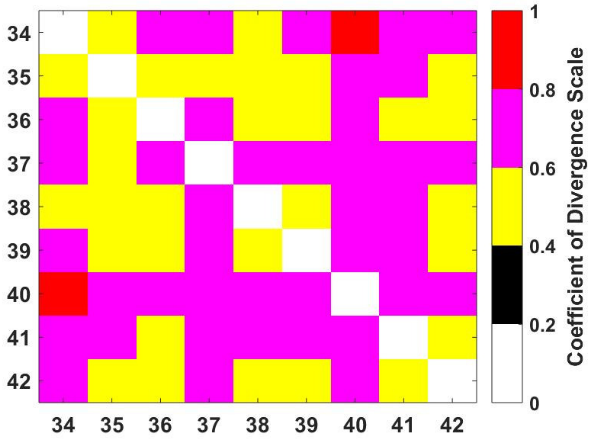

Figure S5. Variability of SO_2. The COD plot for SO_2 concentrations at the nine sites near the industrial facilities (Near Steel Mill, East of Coke Plan, and West of Coke Plant) demonstrates that there are significant differences (COD > 0.2) in SO_2 concentration between sites influenced by these point sources.

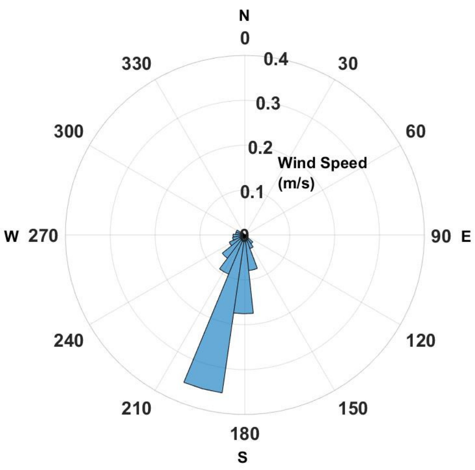

Figure S6. Wind measurements were taken using an RM Young 81000 Sonic Anemometer from January 2018 through December 2018. An exemplary one-month subset of this data is displayed in the wind rose showing one-minute averaged measurements of wind direction and speed. The prevailing wind direction throughout the study domain was from southwest to northeast.
